# Traumatic rosette cataract

**DOI:** 10.1002/ccr3.7834

**Published:** 2023-08-24

**Authors:** Mehrdad Motamed Shariati, Mohammadreza Same

**Affiliations:** ^1^ Eye Research Center Mashhad University of Medical Sciences Mashhad Iran

**Keywords:** amblyopia, blunt trauma, ocular injuries, rosette cataract

## Abstract

One of the clinical presentations of traumatic cataracts is a rosette‐shaped posterior capsular opacity. The severity of vision loss, the patient's age, and the cornea, macula, and optic nerve states are major determinants of the therapeutic approach.

## Graphical abstract

An slit photo of posterior subcapsular rossete‐shaped cataract in a 50‐year‐old woman with a history of ocular blunt trauma of the right eye in childhood.

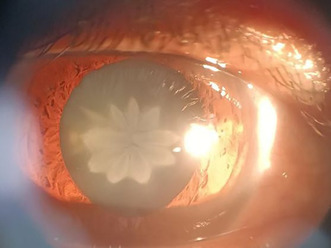

## 1 IMAGE DESCRIPTION

**FIGURE 1:**
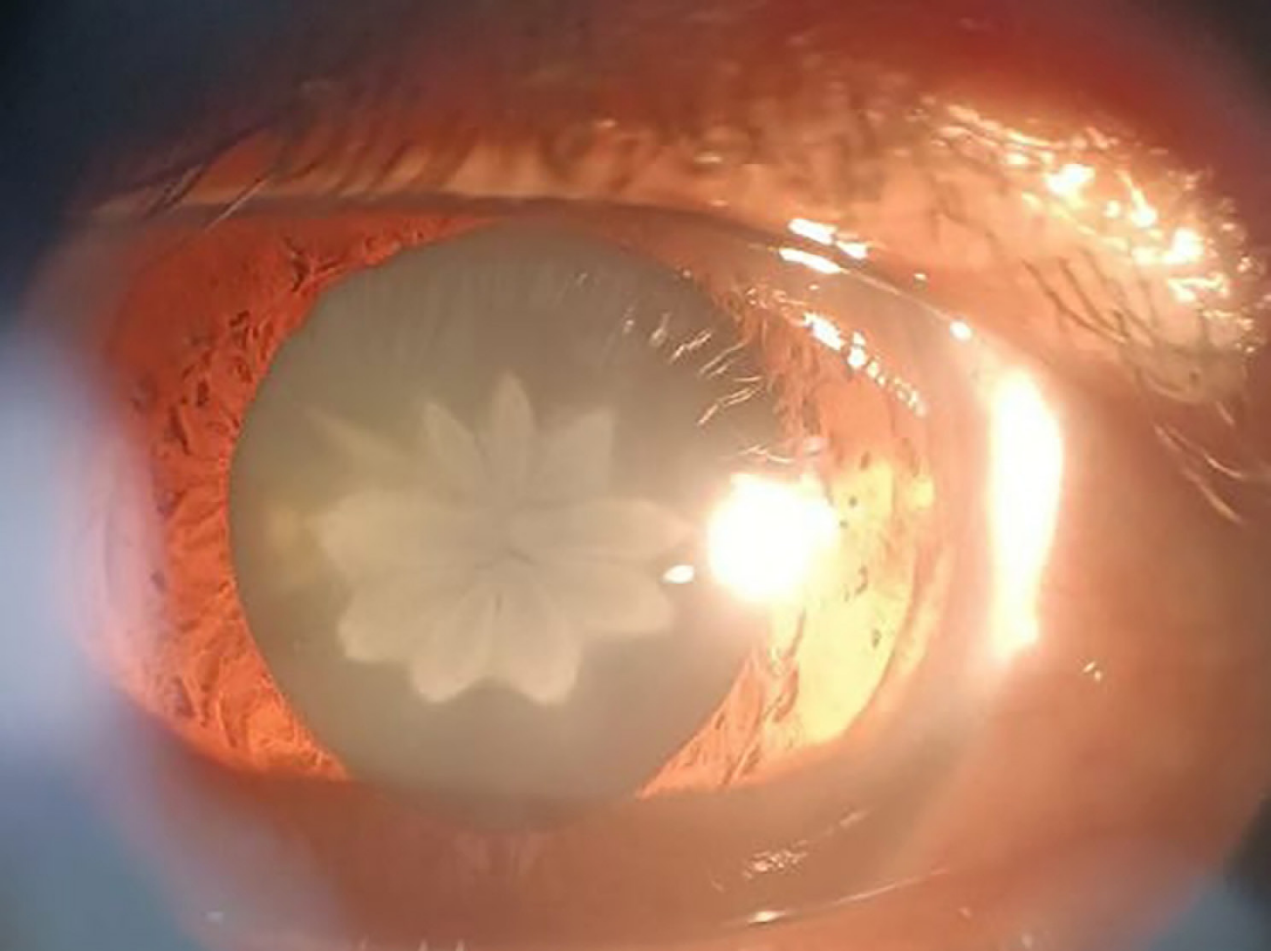
Rosette cataract after blunt trauma.

A 50‐year‐old woman with a history of ocular blunt trauma of the right eye (RE) in childhood was referred to our clinic with blurred vision and glare. In the ophthalmic examination, we found posterior subcapsular rosette‐shaped cataracts (Figure 1). The best corrected visual acuity (BCVA) was counting fingers at 1 meter for the RE and 20/20 for the left eye (LE). Fundus examination and intraocular pressure did not reveal any significant abnormalities in both eyes. The patient's visual impairment had been established since childhood. After explaining that there is a high possibility of amblyopia, the patient did not consent to cataract surgery.

## 2 DISCUSSION

**FIGURE 2:**
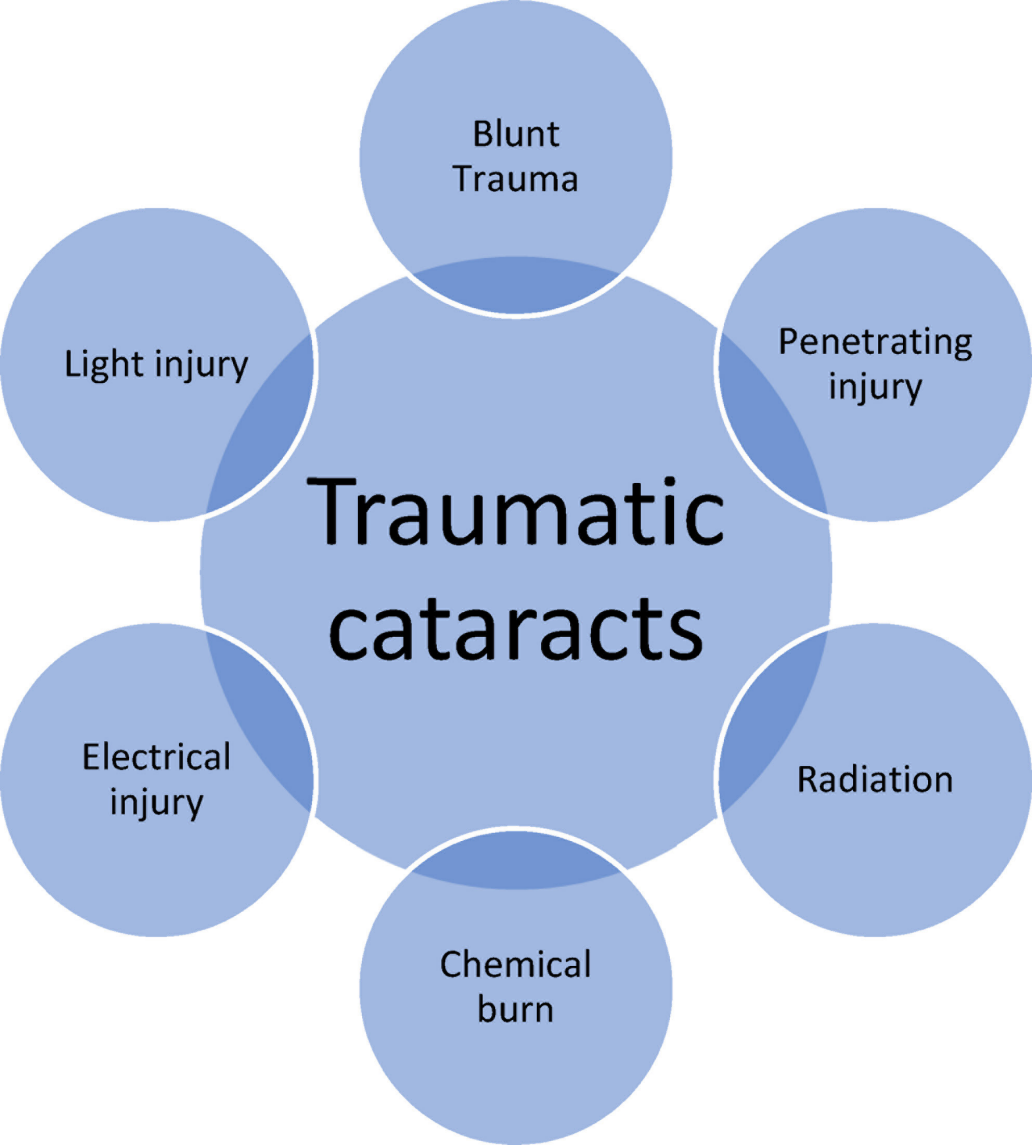
Traumatic cataracts classification considering the mechanism of trauma.

Traumatic cataracts can occur after penetrating or blunt ocular trauma. The damage and lens fiber disruption leads to crystallin lens clouding. Cataract severity, mechanism of trauma, patient's age, and the presence of concomitant ocular injuries influence our therapeutic decision‐making. Cataracts may occur immediately or late after blunt trauma.^1^ Careful follow‐up examinations in patients with mild traumatic cataracts are vital due to the possibility of disease progression and the occurrence of severe vision impairment. The possibility of developing amblyopia in children renders close follow‐up indispensable in preventing unreversible vision loss.^2^ We summarized the etiologic classification of traumatic cataracts in Figure 2. Clinical features of different types of traumatic cataracts could be differentiating (Table 1).

**TABLE 1:** Common clinical characteristics of different types of traumatic cataracts.

Traumatic cataracts	Common clinical characteristics
Blunt trauma	Stellate posterior subcapsular cataracts Cortical opacification
Penetrating injury	Lens capsule rupture Lens material dispersion in the anterior chamber Cortical opacification
Radiation	Posterior subcapsular opacity
Chemical burn	Cortical and posterior subcapsular cataracts
Electrical injury	Feathery cortical opacification
Light injury (ultraviolet, Infrared)	Cortical and posterior subcapsular cataracts

**FIGURE 3:**
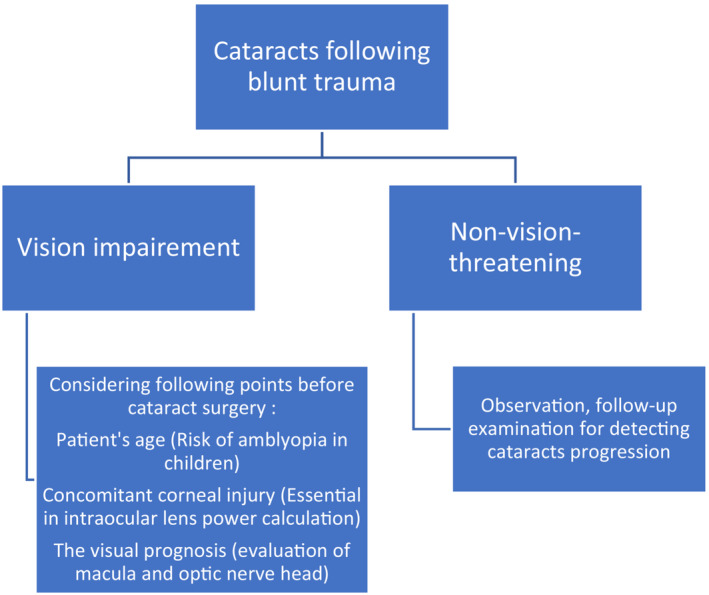
Management approach to a patient with cataracts following ocular blunt trauma.

The therapeutical approach to cataracts following blunt ocular trauma is summarized in Figure 3.^2^, ^3^ Amblyopia is a serious complication in children after traumatic cataracts. Cataract surgery prevents permanent vision loss in these cases.^1^

## AUTHOR CONTRIBUTIONS

**Mehrdad Motamed Shariati:** Conceptualization; supervision; writing – original draft; writing – review and editing. **Mohammadreza Same:** Data curation; investigation; visualization.

## FUNDING INFORMATION

The authors received no funding.

## CONFLICT OF INTEREST STATEMENT

The authors declare that they have no competing interests.

## CONSENT FOR PUBLICATION

Written informed consent was obtained from the patient for the publication of this clinical image report. A copy of the written consent is available for review by the Editor‐in‐Chief of this journal.

## Data Availability

The datasets used during the current study are available from the corresponding author upon reasonable request.
